# Meetings of Societies

**Published:** 1900-12

**Authors:** 


### MEETINGS OF SOCIETIES.

### Bristol Medico-Chirurgical Society.

*Annual Meeting, October 10th, 1900.*

Dr. D. S. Davies, President, in the Chair.

After a vote of thanks to the retiring President (Mr. W. H. Harsant) had been unanimously passed, Dr. D. S. Davies (the President) took the Chair, and gave his introductory address (see page 289), for which a cordial vote of thanks was given him.

The Honorary Secretary (Mr. J. Paul Bush) read his Annual Report. He had to record that the members of the Society had lost two of their number through death; viz., Drs. J. Broom and W. F. Carter. The balance in hand was £16 15s. 6d., together with £97 16s. 1d. on the *Journal* account. He thanked Mr. G. Munro Smith for acting as Secretary during his seven months' absence in South Africa. This report was adopted.

The Editorial Secretary (Mr. James Taylor), in his report for the year 1899, stated that the profit made by the *Journal* was 18s. 2d.

The Honorary Librarian of the Medical Library (Mr. L. M. Griffiths) gave his annual report, and stated that the Society's Library contained 8,741 volumes and 189 periodicals, and that the Bristol Medical Library, of which the former was a part, contained 19,750 volumes and 226 periodicals.

Mr. C. H. Walker showed a patient with sub-conjunctival dislocation of the lens.

The following officers were chosen for the ensuing year: President-elect, Dr. Barclay J. Baron; Honorary Secretary, Mr. J. Paul Bush; Honorary Librarian, Mr. L. M. Griffiths; Members of Committee,

Dr. J. Michell Clarke, Mr. J. Dacre, Dr. B. M. H. Rogers, Mr. G. Munro Smith, Dr. H. Waldo, and Mr. J. Taylor; Members of the Library Committee, Mr. L. M. Griffiths, Mr. G. Munro Smith, and Mr. J. Taylor.

---

*November 14th, 1900.*

Dr. D. S. Davies, President, in the Chair.

Dr. A. B. Prowse showed a specimen of cardiac embolism. The patient from whom it had been obtained, after showing no grave symptoms for fourteen days, had been seized suddenly with cardiac pain and died very soon after. The clot almost completely blocked the pulmonary artery, and was thought to have probably come from a thrombus in the femoral vein.

Dr. Michell Clarke showed four cases. Case 1 was one of progressive muscular atrophy, peculiar in that the wasting had begun in the shoulder. Case 2, he thought, was one of tumour in the interpeduncular space. The case had been shown to the Society some six or eight months previously. The diagnosis was not then so clear. Intentional tremor in the left arm was a marked symptom. This symptom was occasionally seen in tumours of the brain. It had been found in cases of tumour of the optic thalamus, and of the cerebellum. Case 3 was one of tumour in the motor area of the brain. The illness had begun with a fit of general epileptic type in December, 1896. She had a number of these fits in 1897. Later they changed in type and began with sensations in the left foot and leg. The leg became drawn up, then the body was turned over to the left and the left arm drawn up. The spasms began as twitchings, followed by tonic contractions. Several times when the patient had thought a fit was coming on, the ward sister had apparently checked its onset by speaking sharply to her. This might have led to a diagnosis of hysteria. There was no paralysis at this time, but the knee-jerks and elbow-jerks were exaggerated. In May, 1899, the fits had become infrequent; there was no optic neuritis, and very little headache. She could not easily or quickly let go her grasp of any object. Now she had left hemiplegia and constant spasm, and marked optic neuritis and imperfection of vision. Case 4 was a boy aged 8 years. He had been well until 7 years of age. Since then there had been progressive mental deterioration. His mother had had two brothers who became paralysed at about 10 years of age. The boy could hardly stand, and tended to walk and fall backwards. He had spastic paraplegia of legs and arms, and thick and indistinct speech. There was no optic neuritis, but he had become completely blind in the last three or four months. The case was considered to be one of cerebellar tumour.—Dr. Stack asked if Dr. Clarke had ever before found such a degree of spasticity as this boy presented in cases of cerebellar tumour. He would have thought a diffuse cortical lesion, *e.g.* a sclerosis or simple meningitis, more likely to produce the symptoms presented, and alluded to a case he had seen in which cirrhosis of the liver in a young patient was associated with cerebral sclerosis. Other cases had been recorded of this association which had not, as yet, been explained.—Dr. Michell Clarke replied that the diagnosis in case 4 was quite open to question. It might be a case of degeneration of the brain cortex, but that diagnosis would not easily account for the spasticity and tendency to fall backwards.

Mr. C. A. Morton showed two patients, one with congenital varix of the left lower limb, perineum and penis, and the other with congenital dislocation of the hip. A skiagram of the latter was also shown.

Dr. A. OGILVY showed two patients with congenital anomalies of the eyes. In one the iris was absent; in the other there were synechiæ, a polar cataract, &c., the result of intra-uterine inflammation.

Dr. THEODORE FISHER showed a child with congenital stenosis of the pulmonary artery. There was a loud rumbling and rattling bruit heard over a localised area on the left side of the sternum. No clubbing of the fingers, thrill, or cyanosis was present. Occasional faintness was complained of. He mentioned an example of this affection he had seen in a young man of 25 years of age, who came to him to be examined for insurance. In spite of the malformation, the patient could cycle up hills well. Dr. Fisher also showed the following specimens:—(1) A heart with the aorta and pulmonary artery arising from the right ventricle; (2) A heart with the aorta arising from the right ventricle and the pulmonary artery from the left ventricle.—Dr. J. O. SYMES thought the case might be one of patent ductus arteriosus, as there was so little dilatation of the right side of the heart, and no thrill nor cyanosis.—Dr. MARKHAM SKERRITT agreed with the diagnosis of pulmonary stenosis. There appeared to be very little mingling of arterial and venous blood, and one would reason therefore that the obstruction was not great, and that would explain the slight degree of enlargement of the right heart.—Dr. FISHER, in replying, remarked that the murmur in cases of patent ductus arteriosus was diffused, but in his case was very localised on the left side.

Dr. JAMES SWAIN showed the following specimens from aged patients:—(*a*) and (*b*) multiple calculi removed by supra-pubic cystotomy; (*c*) a columnar epithelioma removed by colectomy. In the last case the affected portion of the colon had been brought outside the abdomen at the first operation by the method of Reclus, and later removed by a second operation. A fistula remained, which he had attempted to close unsuccessfully; but the patient was very comfortable notwithstanding. Dr. Swain remarked that he had found old people bore surgical operations well; but he was convinced that it was of great importance to avoid shock by operating quickly, and to avoid hypostatic pneumonia by getting them up early.—Mr. C. A. MORTON mentioned the case of a woman of 70 from whom he had removed both breasts by two operations, a woman of 73 from whom he had removed a large breast tumour, and a man of 70 in whom he had crushed a vesical calculus. All the cases recovered well.—Mr. EWENS alluded to cases of successful operations on the aged in his own practice. —Dr. STACK mentioned the case of a man of 100 years of age who had borne well a minor surgical operation.

Mr. G. MUNRO SMITH showed a skiagram (and a drawing) of the injury known as "stave of the thumb." He explained that the term was applied to an impacted fracture of the base of the metacarpal bone of the thumb, in which the small fragment was split vertically through the pressure on the trapezium, as well as broken off transversely. Two other examples of this injury were alluded to. The patient had but slight limitation of movement in consequence of the injury. Mr. Smith also showed the larynx of a child who had been suffocated through the impaction of a piece of walnut in the larynx just below the vocal cords.

Dr. E. A. H. GROVES read a paper on "Eclampsia." The notes of two cases which had been successfully treated by saline intra-venous transfusion were read. The first was that of a primipara who gave birth to a child at 5 a.m. She became convulsed when the placenta was expelled. There was little or no hemorrhage. The fits recurred every hour. Morphia and pilocarpine were first tried, but she became worse. Breathing was stertorous, the pulse 120. No urine was found on catheterization. At 5 p.m. a hundred ounces of saline fluid were transfused, the proceeding taking fifty minutes. At first there was trouble with the blood coagulating in and around the cannula. During the next three hours there were two slight fits; there had been eight in the previous twelve hours. Urine began to be secreted in twenty-four hours. The second case had many convulsions before labour was completed, and became absolutely comatose. A few drachms of urine found in the bladder were solid with albumen. The os was dilated with Champetier's bag and delivery completed. Fifty ounces of saline fluid were injected. The same difficulty with clotting was met with as in the other case. Improvement set in immediately after the transfusion. Dr. Groves then discussed the pathology of eclampsia, and the mode of action of saline injections. He remarked that no constant gross lesions were found in eclampsia. But recent work showed that there were analogous changes in the brain, liver, lungs, and kidneys. Multiple minute thrombotic and hemorrhagic areas were found in these organs. The eclamptic kidney was rather of a degenerated than of an inflammatory type. The liver lesions had been compared to those in acute yellow atrophy. The blood was described as thick and dark and of greatly increased coagulability. The right side of the heart was said always to contain coagula in the fatal cases, in spite of the asphyxia which tended to maintain the fluidity. The literature referring to the use of saline fluid in the treatment of eclampsia was alluded to. The first cases the speaker had found recorded were in 1893 and 1894. In a total number of 47 cases in which this treatment was used, 41 recovered and 6 died. The action probably was that the dilution of the blood hindered further thrombosis and helped to dissolve thrombi already formed. The relief was not due to diuresis. Diuresis was rather a result than a cause of the relief. Relief occurred immediately, before diuresis was established.—Dr. Aust Lawrence said that in these cases the first thing to be done was to deliver as quickly as possible. He thought saline injections were very useful, whether given *per* rectum, or by the skin, or through the veins. Injection under the skin required less skill, and was more applicable when assistance was not at hand. A couple of pints could be given subcutaneously. The swelling formed disappeared in a few minutes. Eclampsia could be prevented in many cases if the premonitory symptoms were recognised; viz., swelling of the feet, headache, dimness of vision, &c., and if on their appearance the patients were put to bed and kept on a milk diet with citrate of potash as medicine. He alluded to a case in which veratrum viride had acted very beneficially. The patient had a bounding and hard pulse of 120. Ten minutes after giving ♏ v. of the liquid extract of veratrum the pulse was 60. If the pulse gets too low brandy or ether raises it again. Saline injections, he remarked, were not going to take the place of all other drugs. He had seen a case in which morphia seemed to save life.—Dr. Temple asked why bleeding benefited the cases, seeing that the blood was viscous.—Dr. Walter Swayne agreed that saline fluid was a very valuable remedy. He usually gave it *per* rectum with a long tube. One method of treatment was not suitable for all cases. He hoped for *post partum* hemorrhage in these cases, but it never came. When cyanosis and turgidity of the face existed he regarded venesection as probably the best treatment. The blood became more watery after venesection, which explained Dr. Temple's difficulty. He thought intra-venous injection too drastic in many cases. It was simpler to inject the fluid into the submammary or subcutaneous tissues with a syringe. Rectal injections were the simplest of all; but the tube had to be held in all the time, while some of the fluid escaped from the anus.—Dr. GROVES, in replying, said there was a notorious difference of opinion about all the remedies which were used except saline fluid, which everyone had found beneficial, and pilocarpine, which everyone decried. The saline fluid was not simply a stimulus, but was a direct remedy attacking the source of the disease.

At the close of the meeting Mr. J. PAUL BUSH showed a series of lantern slides illustrating scenes during the Boer War.

J. LACY FIRTH, *Reporter.*
J. PAUL BUSH, *Hon. Sec.*

